# MRI-guided detection of knee injuries as a concomitant lesion of femoral shaft fractures: a systematic review and meta-analysis

**DOI:** 10.1530/EOR-2025-0184

**Published:** 2026-07-01

**Authors:** Tayfun Yilmaz, Guido Schwarzer, Andreas Fuchs, Markus Siegel, Nils Mühlenfeld, Ferdinand C Wagner, Hagen Schmal, Kaywan Izadpanah, Andreas Frodl

**Affiliations:** ^1^ Department of Orthopedics and Traumatology, Freiburg University Hospital, Freiburg, Germany; ^2^ Institute of Medical Biometry and Statistics, Faculty of Medicine and Medical Center – University of Freiburg, Freiburg, Germany; ^3^ University Hospital Odense, Department Of Orthopedic Surgery, Odense C, Denmark

**Keywords:** knee injury, femoral fractures, incidence, ligaments

## Abstract

- *Purpose:* The aim of this study is to identify possible predilection sites for ligamentous injuries of the knee joint and, based on these findings, provide recommendations to reduce the risk of missed injuries of the knee in cases of ipsilateral femoral shaft fractures.
- *Methods:* We conducted a systematic review searching the Cochrane, PubMed, and Ovid databases. Inclusion criteria were the modified Coleman methodology score (mCMS) > 50, fully grown patients with mature skeletal development, with ipsilateral femoral shaft fracture and MRI imaging of the knee to assess ligamentous and meniscal injuries objectively. Skeletal immature patients, biomechanical or cadaveric studies, pathological fractures or periprosthetic fractures and animal studies were excluded.
- *Results:* We analyzed the data of 636 patients out of 4 manuscripts regarding ligamentous knee injuries with ipsilateral femoral shaft fractures. The collateral ligaments, especially the medial collateral ligament (MCL) is seen with the highest injury rate of 23% (95% CI: 9–41%) followed by the lateral collateral ligament with 10%. The injury-rate was calculated with 9% (95% CI: 4–13%) for the anterior cruciate ligament and 7% (95% CI: 0–19%) for the posterior cruciate ligament.
- *Conclusion:* Concomitant ligamentous knee injuries are common in femoral shaft fractures and should be suspected especially in trauma mechanism involving vehicle collisions. Systematic post-stabilization knee examination and with suspected ligamentous injury a timely MRI, with referral to higher-level centers when unavailable, may reduce missed injuries and improve outcomes.

## Introduction

Femoral shaft fractures are typically caused by high-energy trauma mechanisms, in which, due to the blunt force, concomitant injuries to adjacent joints are likely (1, 2, 3). The risk of concomitant, ligamentous knee injuries in patients with ipsilateral femoral shaft fractures was first described by Pedersen and Serra 1968 (4). Joint stability was assessed by physical examination. Given the significant pain experienced by patients resulting from the malalignment caused by a fracture, manual examination of the injured limb is however limited before fracture stabilization (5, 6). After the stabilization of the fracture, a manual examination is indeed more feasible, but even in this case, both the execution of the examination and its assessment are heavily dependent on the examiner and thus give potential to miss relevant ligament injuries. These injuries, in particular, can cause a delay in the rehabilitation phase and promote the development of early osteoarthritis, if not detected early and treated appropriately (7).

In this regard, arthroscopic procedures and recently MRI have been employed as diagnostic tools for better tracking and objectification of knee injuries (8, 9).

Data concerning ligamentous knee injuries following femoral shaft fracture are scarce. Especially when MRI diagnostics have been applied.

Based on existing literature, this study aims to address the following research questions:

- Is there a statistically significant number of knee joint injuries associated with ipsilateral femoral shaft fractures?
- In cases of femoral shaft fractures, is there a relative accumulation of injuries to specific ligamentous structures in the ipsilateral knee?
- Can recommendations for clinical practice be derived from the results of this study?

## Methods

This systematic review was conducted adhering to the guidelines of the Preferred Reporting Items for Systematic Reviews and Meta-Analyses check list (PRISMA) (10). The study protocol was registered in PROSPERO (ID: CRD42024499705.), a database listing current meta-analyses, at the beginning of our literature search.

The authors AF and TY independently conducted a database search. Studies published until 01.02.2024 were checked for eligibility. MEDLINE, PubMed, Embase and the Cochrane Central Register of Controlled Trials (CENTRAL) were searched for relevant studies reporting ligamentous knee injuries in the context of ipsilateral femoral shaft fractures. Additionally, reference lists of potentially eligible publications were scanned to asses if relevant records were missed by the search algorithm.

To calculate the risk of underlying bias, all included studies were analyzed with the Newcastle–Ottawa Scale (NOS) and visualized using the RobVis-tool (11, 12).

### Search strategy

The following search strategy was applied, limiting the year of search by the start of 1990, when MRI-diagnostics were used more commonly: ((((femoral shaft) OR (femoral diaphysis)) AND fracture) AND ((knee injury) OR (knee lesion) OR (ligament) OR (meniscus)))

### Eligibility

We applied the following inclusion criteria: fully grown patients with mature skeletal development, with ipsilateral femoral shaft fracture. MRI imaging of the knee in over 80% of the study population to assess ligamentous and meniscal injuries objectively. Only publications written in German or English were included. Our exclusion criteria were: an overall modified Coleman Methodology Score (mCMS) < 50, skeletal immature patients, biomechanical or cadaveric studies, pathological fractures or periprosthetic fractures. Combined fractures of the tibia and femur, also classified as floating knee according to Fraser, were also excluded (13).

According to the aforementioned inclusion and exclusion criteria the same reviewers independently screened titles and abstracts for relevance. If no abstract was available, the full-text was obtained to assess the study’s relevance. To make sure not to overlook any suitable studies, we cross-referenced the references within included articles if they were missed by our search algorithm. Appropriate publications were then independently analyzed for the mCMS and level of evidence according to the Oxford Centre of Evidence-Based Medicine (14).

### Outcome criteria

Patient demographics, number of patients, number of patients with ligamentous injuries, type of ligamentous injury and number of meniscal injuries were extracted (Table 1A and B).

**Table 1:** Demographic overview of included studies (A) and overview of the number of ligamentous injuries (B).

	Blacksin *et al.* (19)	Dickson *et al.* (20)	Byun *et al.* (18)	Dawood *et al.* (5)
A) Study characteristics				
Study type	PS	PS	RS	PS
mCMS-score	58	55	67	67
Level of evidence	III	III	III	III
Patients, *n*	34	27	429	146
Age, years	27.0	NI	40.7	NI
Occurrence of ligamentous IKI	24	26	87	54
B) Ligmentous injuries, *n*				
Medial collateral ligament	13	11	34	24
LCL	2	8	24	12
ACL	2	5	29	18
PCL	7	2	42	0
Meniscal tears	10	11	NI	0

RS, retrospective study; PS, prospective study; IKI, ipsilateral knee injuries; LCL, lateral collateral ligament; ACL, anterior cruciate ligament; PCL, posterior cruciate ligament.

### Statistical analysis

Analyses of ligamentous injuries in general and the type of ligamentous injury were performed by GS. Random-effects meta-analyses were conducted to estimate the pooled ligament injury rates. Injury rates were arcsine transformed before pooling and the restricted maximal likelihood (REML) estimator for the between-study variance was used (15). *I*^*2*^ was calculated to assess the magnitude of between-study variation (16). Meta-analysis results were visualized in forest plots. Meta-analyses were performed in R, version 4.3.2, using the meta package (Version 4.3.2, the meta package, Vienna, Austria, the R-foundation, 2022) (17).

## Results

### Study selection

**Figure 1:**
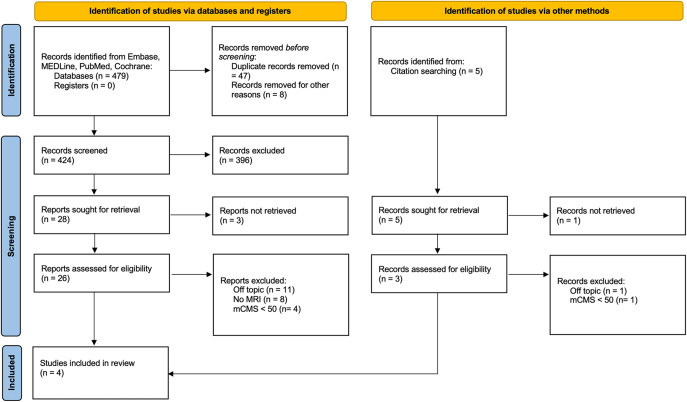
Flow chart of study selection process according to the PRISMA 2020 statement; note: mCMS: modified Coleman Score.

Our literature search and study selection procedure is depicted in Fig. 1, and a total of 479 papers were identified by our search algorithm. Moreover, we added two papers listed in the references in one of the eligible publications.

All papers were scanned. Any duplicates or topic-unrelated articles were excluded. After analyzing the eligibility criteria, four studies could be included in our quantitative analysis (5, 18, 19, 20). There were three prospective studies and one retrospective study containing a total of 636 patients. The number of patients included in the selected studies ranged from 27 to 429 with a mean age of 33.8 ± 6.8 years.

### Risk of bias assessment

**Figure 2:**
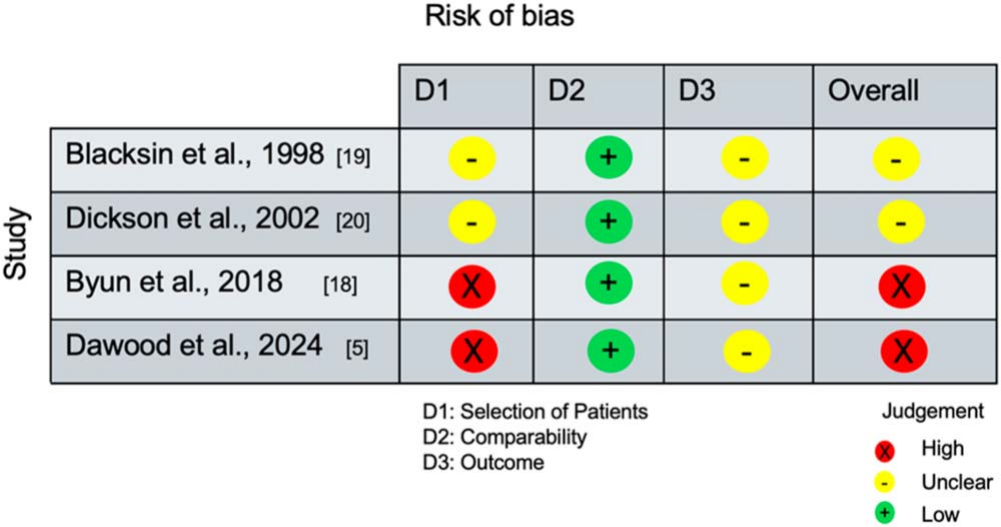
Risk of bias assessment with Newcastle Ottawa scale (NOS) visualized with RobVis tool.

All included studies possessed an evidence-level III. Reporting and detection biases are considerable due to the lack of randomization and blinding. Considering the retrospective design of one of the included studies, there is a high risk for selection bias. Our results for the risk of bias assessment are shown in Fig. 2.

### Occurrence of ipsilateral, ligamentous knee injuries

**Figure 3:**
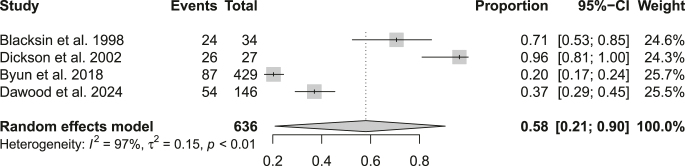
Forest plot of the overall rate of ligamentous knee injuries in ipsilateral shaft fractures of the femur.

Ipsilateral, ligamentous injuries to the knee occurred in 191 patients resulting in a weighted occurrence rate of 58% (95% CI: 21–90%) with considerable heterogeneity (I-squared = 97%) (Fig. 3).

### Injury to the cruciate ligaments

**Figure 4:**
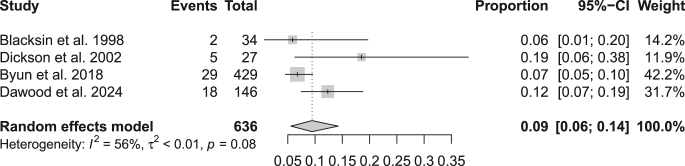
Forest plot of injury-rate of ACL.

**Figure 5:**
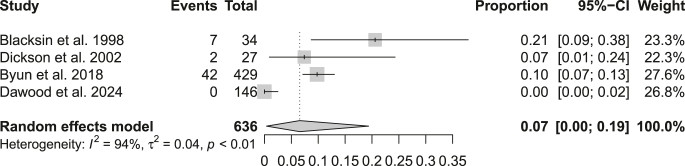
Forest plot of injury-rate of PCL.

In a sub-analysis we assessed the injury rate of cruciate ligaments. For analysis the total number of torn cruciate ligaments was extracted. There was a total of 51 (8.0% of the study population) injuries to the PCL (posterior cruciate ligament) and 54 (8.5%) to the ACL (anterior cruciate ligament). The injury-rate was calculated with 9% (95% CI: 6–14%) for the ACL and 7.0% (95% CI: 0–19%) for the PCL. Results are depicted in Figs 4 and 5.

### Injury to the collateral ligaments

**Figure 6:**
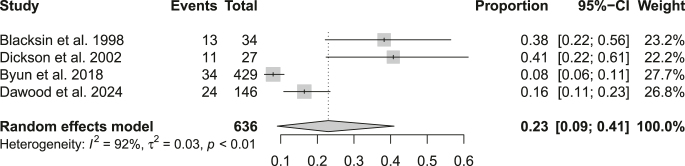
Forest plot of injury-rate of MCL.

**Figure 7:**
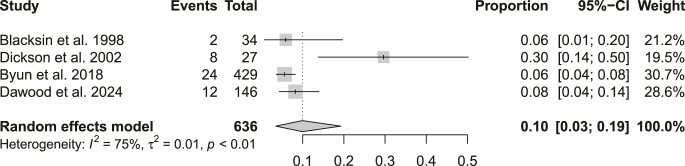
Forest plot of injury rate of LCL.

Likewise, an analysis for lesions to the collateral ligaments was performed. Overall, the total number of injured MCL (medial collateral ligament) was higher than with LCL (lateral collateral ligament). Injury rates were calculated with 10% (95% CI: 3–19%, *I*^2^ = 75%) for the LCL and 23% (95% CI: 9–41%, *I*^2^ = 92%) for the MCL (Figs 6 and 7).

### Meniscal injuries and tears

**Figure 8:**
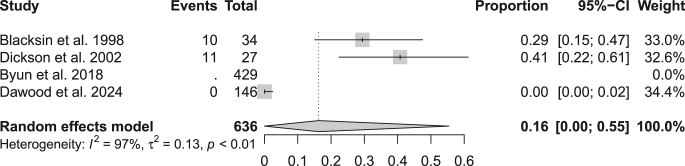
Forest plot of rate of meniscal lesions.

Data to 21 concomitant meniscal injuries were extracted from Dickson *et al.* and Blacksin *et al.* (19, 20). Thus, an injury-rate of 16% (95% CI: 0–55%, *I*^2^ = 97%) was calculated (Fig. 8). There were no documented data concerning the meniscus with Byun *et al.* and Dawood *et al.* (5, 18).

## Discussion

Femoral shaft fractures are often caused by high impact trauma. As a result, concomitant injuries, especially to the knee joint, are likely.

However, regarding the type and number of injuries, there are very wide-ranging data available in the literature so far. This is mainly due to the different diagnostic methods used for diagnosis assessment or confirmation. There are studies that solely use physical examination for diagnosis, as well as studies that supplement it with either arthroscopy or MRI (4, 9, 18).

In this systematic review, we could show that concomitant injuries to the knee joint are a common entity in femoral shaft fractures. The main results of this study are: the medial colateral ligament (MCL) complex is injured the most. Injuries to the ACL and LCL are affected less often followed by lesions to the PCL.

Femoral shaft fractures are a common injury in high-impact trauma, such as car accidents. They occur with an incidence of 10–21/100,000 people (21, 22). Initial examination of the adjacent joints, especially the knee joint, is difficult or even impossible. An examination is only possible after fracture stabilization. A standardized examination algorithm should therefore be established so that knee injuries are not missed.

In 1968, Pedersen and Serra were the first to describe a connection between fractures of the femoral shaft and internal knee structures (4). These observations were subsequently confirmed. Initially, diagnosis was based solely on clinical examination results, but later on arthroscopic and MRI findings were also included in the assessment.

There are risk factors for the occurrence of concomitant injuries to the knee joint with accompanying femoral shaft fractures. As risk factors, both the type of fracture and the trauma mechanism itself can be considered.

The probability of concomitant internal knee injuries rises in proportion to the severity of femoral shaft fractures. Type B and Type C fractures according to the AO classification, and especially traffic accidents, are significant risk factors for a potential knee joint ligament injury (18).

Despite knowledge of the risk factors and the possibility of concomitant injuries to the knee joint in ipsilateral femoral shaft fractures, a delay in diagnosis is possible in routine clinical practice, particularly in polytraumatized patients.

The probability of the occurrence of injuries that are not detected in the emergency department can be estimated based on certain risk factors. The following factors are listed in descending order of probability: 2 or more body regions are affected, initial Glasgow Coma Scale 3–8, small and medium sized trauma centers, Blood transfusion required before ICU admission, cardiac arrest or CRP, motorbike rider, car passenger and 6 or more injuries identified in the emergency department (23).

In addition to focal clinical findings such as joint effusion or hematoma, the clinical examination of the knee joint was the key determinant for MRI indication in the studies included. The first reliable assessment of the knee joint was performed after stabilization of the femoral fracture, typically during an examination under anesthesia. When this examination suggested ligamentous injury, further evaluation by MRI was undertaken to proof the findings of physical examination.

However, the timing of MRI diagnostics varied among the included studies. MRI was performed either early in the postoperative period or selectively during the subsequent clinical course (18, 19). In particular, the study by Byun *et al.* reported the longest interval to diagnostic confirmation by MRI, with a mean time of 10.6 weeks, whereas Blacksin *et al.* demonstrated the shortest interval, with MRI performed on average 2.5 days after injury. which may potentially be explained by the retrospective study design of Byun *et al*.

This heterogeneity underscores the need for standardized diagnostic algorithms and clearly defined indications for MRI in patients with femoral shaft fractures.

In addition to the specific factors that increase the risk of a knee injury in the case of a femur fracture, the general influencing factors should also be considered.

Delayed diagnosis and therefore delayed treatment can lead to a poorer clinical outcome with persistent instability and therefore an early onset degeneration of the knee (24, 25).

When considering ligament injuries separately in relation to the underlying pathomechanism, the mechanisms of injury can be broadly categorized into ‘direct impact trauma’ and ‘dynamic trauma’. The former includes high-energy impacts resulting in valgus or varus stress, as well as PCL ruptures in the form of a dashboard injury. Deceleration injuries with direct impact (e.g. dashboard injury or vehicle–pedestrian collision), as well as excessive valgus stress and hyperextension, are described as key pathomechanisms for ligamentous injuries of the knee joint (26).

Therefore, the mechanism of injury represents a crucial factor, as its kinematic characteristics alone may suggest the presence of ligamentous damage (27).

The studies included in this analysis initially evaluated the mechanism of trauma as a determinant of the likelihood of internal derangement of the knee joint. In particular, road traffic accidents and falls from height were identified as major risk factors (5, 18, 19, 20). Road traffic accidents included both incidents in which patients were injured as drivers or passengers and those in which patients were struck as pedestrians by a vehicle.

Asgari and Keyvanian demonstrated the effects of car-to-pedestrian accidents on knee ligament injuries using anatomically constructed 3D finite element models, which highlighted the MCL as a particularly vulnerable structure in this type of mechanism (28). These purely theoretical models were also corroborated by the registry study conducted by Mallory *et al.* In this study, combined ligament injuries involving both collateral and cruciate ligaments were particularly evident. The occurrence of isolated cruciate ligament ruptures appeared to be dependent on the magnitude of the force applied, which was exerted either just above or below the knee, in conjunction with anterior-posterior loading (29).

Due to the different sensitivities of the tests for the respective structures, an exclusively clinical diagnosis is also associated with a corresponding rate of missed injuries or delayed diagnosis of injuries. For example, the sensitivity for the posterior drawer is very variable and amounts to 50–90% depending on the literature (30, 31, 32, 33).

The clinical diagnosis of ACL has a better sensitivity. The Lachmann test, for example, is between 80 and 98%, whereby the examination under anesthesia has the better sensitivity (34, 35, 36, 37).

However, there are large disparities in the testing of MCL and LCL. The sensitivities here are between 45 and 96% (33, 38, 39, 40, 41).

Additionally, after fracture treatment, an examination of knee ligament stability can be performed through direct side-by-side comparison. To objectify the results of these examinations, X-ray fluoroscopy similar to stress imaging can also be used. Furthermore, postoperatively, standardized procedures for ligament testing can be employed to confirm any suspicion of knee ligament injuries (42, 43). The safest method for detecting internal knee damage, including ligament injuries, is provided by MRI, which offers the possibility to identify injuries not only early but also in time for timely treatment (44, 45).

Based on this study we were able to demonstrate the frequency of concomitant ligamentous knee injuries. We recommend first analyzing for potential risk factors pointing to ligamentous knee injuries. This starts with the trauma mechanism. Vehicle accidents and pedestrian-vehicle collisions should rise awareness to potential concomitant knee injuries. Although an exact clinical examination of the knee joint may not be feasible at the time of direct admission due to the femoral fracture, a thorough assessment should be performed after fracture stabilization as part of an examination under anesthesia. If this evaluation reveals signs of ligamentous injury, MRI imaging should be performed in a timely manner to allow for prompt identification and management of the associated injuries. If a MRI is not available at the treating trauma center, transfer of the patient to a trauma center with higher level should be initiated.

Thus, the rate of missed injuries can be reduced and the outcome of patients improved.

### Limitations

Limitations of this publication arise out of the type of included studies as well as our search algorithm. The risk of publication bias is imminent because only published articles were included. To minimize this kind of bias, the Cochrane Library® was scanned for clinical trials, but we detected no relevant findings, as the results of several ongoing trials have not been published yet.

Our search strategy followed an English search algorithm. Thus, potentially suitable publications in other languages were not considered.

All of the included publications are either retrospective or prospective studies entailing a high risk for selection, detection, and reporting bias. To exclude methodologically inadequate studies, we focused on bias-assessment as done by NOS and mCMS. There was no critical risk of bias in any included study.

Given the substantial heterogeneity in the included data, meta-analysis results have to be interpreted with caution. In this case, it is particularly noteworthy to highlight the study by Dawood *et al.* (5). Unlike the other included studies, no multiligament injuries or PCL lesions were identified in this study. These results need to be critically evaluated in the context, even though an assessment of the study methodology by Dawood *et al.* did not reveal any apparent sources of error.

## Conclusion

Ligamentous injuries of the knee joint are common accompanying injuries in ipsilateral femoral shaft fractures. Trauma mechanism, particularly motor vehicle accidents and pedestrian–vehicle collisions, should be considered a key risk indicator for associated knee injuries. Although comprehensive knee assessment may be limited at initial admission, a systematic examination after fracture stabilization under anesthesia is essential. In cases of suspected ligamentous injury, timely MRI should be performed, and referral to a higher-level trauma center should be considered when MRI is unavailable. Implementing such structured diagnostic pathways may reduce missed injuries and improve functional outcomes.

## ICMJE Statement of Interest

The authors do not have any conflict of interests regarding this study.

## Funding Statement

We acknowledge support by the Open Access Publication Fund of the University of Freiburg.

## Data availability

All data are within the manuscript.
